# Proteomic and extracellular particle changes in preterm neonates with suspected late-onset sepsis

**DOI:** 10.1016/j.isci.2026.117895

**Published:** 2026-10-15

**Authors:** Daniel O’Reilly, Lauren Murphy, Ella Fouhy, Laura Ryan, Claire Anne Murphy, John O’Loughlin, Fionnuala Ní Áinle, Isobel Claire Gormley, Kieran Wynne, Rosanna Inzitari, Peter Doran, Richard J. Drew, Craig N. Morrell, Carmel Maria Moore, Anna Curley, Naomi McCallion, Patricia B. Maguire

**Affiliations:** 1 Conway-SPHERE Research Group, Conway Institute, School of Biomolecular and Biomedical Science, University College Dublin, Dublin 4, Ireland; 2 Department of Neonatology, Rotunda Hospital, Parnell Square, Dublin 1, Ireland; 3 Neonatal Unit, National Maternity Hospital, Dublin 2, Ireland; 4 The Coombe Hospital, Cork Street, Dublin 8, Ireland; 5 Department of Laboratory Medicine, Rotunda Hospital, Parnell Square, Dublin 1, Ireland; 6 Department of Haematology, Mater Misericordiae University Hospital, Dublin 7, Ireland; 7 Department of Haematology, Rotunda Hospital, Dublin 1, Ireland; 8 School of Mathematics and Statistics, University College Dublin, Dublin 4, Ireland; 9 School of Medicine, University College Dublin, Dublin 4, Ireland; 10 Clinical Research Centre, University College Dublin, Dublin 4, Ireland; 11 Clinical Innovation Unit, Rotunda Hospital, Dublin 1, Ireland; 12 Irish Meningitis and Sepsis Reference Laboratory, Children’s Health Ireland at Temple Street, Dublin 1, Ireland; 13 Department of Clinical Microbiology, Royal College of Surgeons in Ireland, Dublin 2, Ireland; 14 Aab Cardiovascular Institute, University of Rochester, Rochester, NY, USA; 15 UCD Institute for Discovery, University College Dublin, Dublin 4, Ireland; 16 Insight Research Ireland Centre for Data Analytics, University College Dublin, Dublin 4, Ireland

**Keywords:** sepsis, neonatal, proteomics

## Abstract

Neonatal sepsis remains an important cause of morbidity and mortality in neonates. In this prospective, multisite observational study, proteomics and extracellular particle analysis were performed on platelet-poor plasma from neonates at the point of clinical suspicion for sepsis (*n* = 110). Neonates were then classified by their clinical teams into three groups: culture-proven sepsis, culture-negative sepsis, or non-sepsis. Neonates with culture-proven sepsis demonstrated statistically significant differences in their analyzed proteome compared to neonates deemed to have non-sepsis, while those with culture-negative sepsis did not. Extracellular particle concentration and type, as determined by flow cytometry, did not differ between the three groups. This study provides further evidence that culture-proven sepsis and culture-negative sepsis are not equivalent diagnoses in the neonatal population without a consensus definition for sepsis. The proteins identified in the culture-positive group may be of utility in determining neonatal sepsis endotypes, but this would require further validation.

## Highlights

• Culture-proven neonatal sepsis is associated with a distinct proteomic profile
• Culture-negative sepsis did not differ from non-sepsis in this cohort
• Extracellular particles did not differ between all three clinical groups
• High IL1RL1/ST2 may indicate risk in neonatal sepsis

## Graphical abstract

**Figure:**
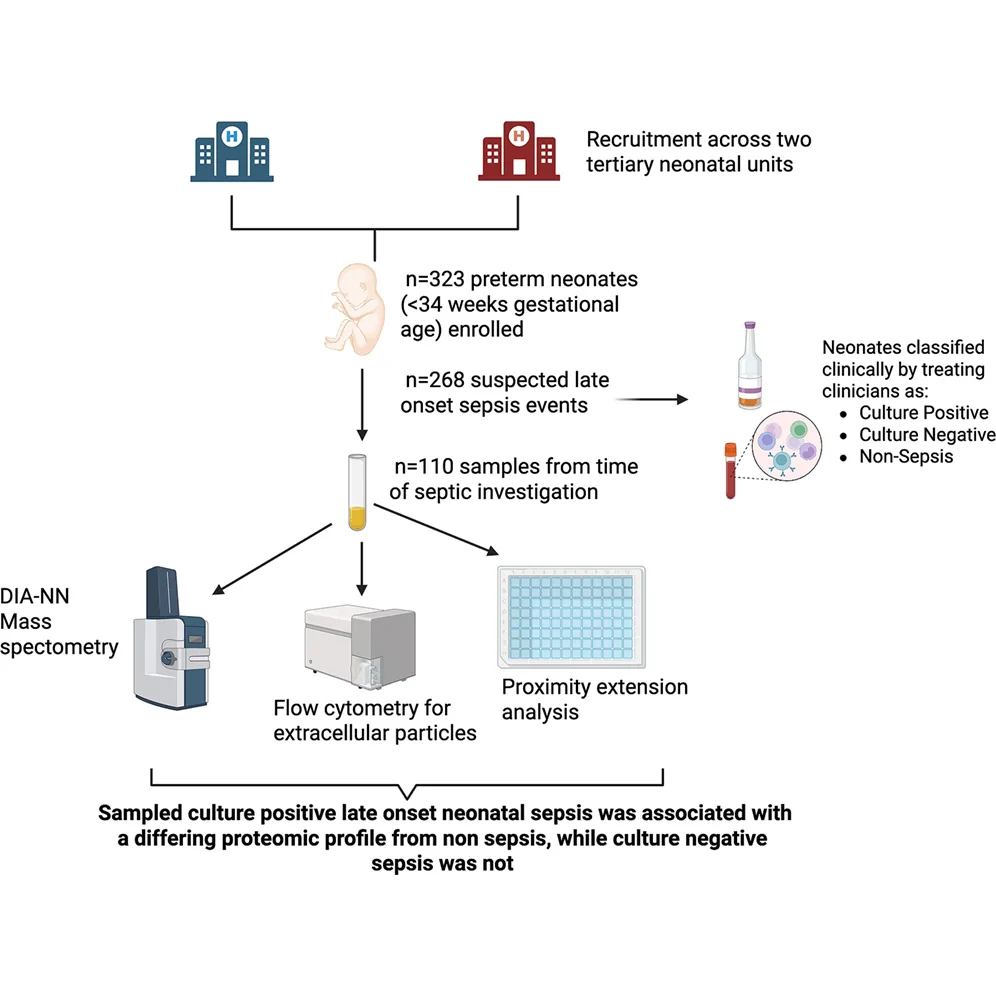

## Teaser

Health sciences

## Introduction

Neonatal sepsis remains an important cause of morbidity and mortality globally. The first 28 days of life are the time of highest risk for the development of sepsis, with an incidence of 6.1/1,000 live births.^1^ The burden of neonatal sepsis is further concentrated in neonates born <34 weeks of corrected gestational age (cGA) with 10% of neonates in this population diagnosed with a blood culture-proven infection, making up over 80% of all identified culture-proven sepsis cases.^2^ This likely represents an underestimate of true bacterial infection, as many significant bacterial infections (SBIs), such as urinary tract infection (UTI), pneumonia, and neonatal meningitis, can occur in the absence of a positive blood culture.^3^

While consensus definitions exist for sepsis in adult and pediatric populations, there is no generally agreed definition of neonatal sepsis beyond the presence of a causative bacterium in a sterile site (typically blood or cerebrospinal fluid).^4^^,^^5^^,^^6^ The high degree of physiological volatility in the highest-risk preterm population has generated difficulty in reaching agreed definitions of organ dysfunction and has consequent challenges for similar definitions of neonatal sepsis.^7^

Given this lack of a consensus definition and the perceived unreliability of blood cultures, many practicing neonatologists prescribe an empiric course of antibiotics for greater than or equal to 5 days based on a combination of clinical findings, signs of inflammation, and/or clinical context.^3^ This is often referred to as “culture-negative” sepsis and is justified based on the finding that a proportion of neonates without “culture-proven” sepsis demonstrated signs of inflammation on postmortem, which could be consistent with infection.^8^^,^^9^ Not only does this represent a classic case of reverse survivorship bias but it also originates with a group of neonates who would have had no access to molecular microbiological diagnostic methods or surfactant and therefore are unrepresentative of modern neonatal cohorts.^8^ A proportion of these neonates may have late-onset viral infections (such as enterovirus and cytomegalovirus [CMV]) that may have an inflammatory response but would not respond to antibiotics.

Previous work has demonstrated that platelets may have a key role in the neonatal response to bacterial infection.^10^ Thrombocytopenia is often considered a poor prognostic sign in neonatal sepsis and is utilized in standardized scoring systems for risk of mortality, such as the neonatal sequential organ failure assessment (nSOFA).^11^ Translational work has demonstrated that, in the context of infection, platelet activation stimulates the bone marrow to produce greater numbers of platelets, which can rapidly lead to marrow exhaustion in the neonatal context.^12^^,^^13^^,^^14^ Studies in animal models have highlighted the role of platelets as auxiliary immune cells.^15^

The markers of platelet activation for identification of late-onset sepsis in infants (PARENT study) was a preregistered, prospective observational cohort study that sought to establish whether platelet and endothelial activation markers in plasma, such as extracellular particles (EPs) and their proteomic contents as well as plasma proteomics, differ in neonatal late-onset sepsis in neonates born at <34 weeks of cGA.^16^ We hypothesized that platelet and endothelial markers in the plasma proteome and EPs by concentration, type, and proteomic content would differ between neonates with culture-proven neonatal sepsis and those who did not have sepsis. As a secondary objective, we examined whether platelet and endothelial markers differed between neonates with culture-negative sepsis and those without sepsis. We performed a final hypothesis-generating cluster analysis to examine differences in groups defined by proteins that differed statistically between clinically defined groups.

## Results

### Patient demographics

A total of 392 families of neonates born at less than 34 weeks of cGA admitted to the neonatal unit were approached, with 323 neonates being consented to future sampling during any investigations for late-onset sepsis (Figure 1). Characteristics of neonates in each diagnostic group and how many were successfully recruited are described in Table 1, while organisms identified in blood cultures in culture-positive sepsis are shown in Table 2. Table 3 demonstrates the characteristics of the group that were sampled with 110 total septic events included, 58 neonates were classified as non-septic, 36 neonates were classified as culture-negative sepsis, and 16 were classified as culture-proven sepsis. Note that neonates with culture-proven sepsis were statistically more likely to be born at a younger gestational age than those with non-sepsis (*p* = 0.03, 95% confidence interval [CI], 0.07–3.2 weeks) but not those with culture-negative sepsis (*p* = 0.06, 95% CI, −3.2 to 0.06 weeks). Culture-proven sepsis was associated with higher vasomotor inotropic score (VIS) and nSOFA scores as well as higher inflammatory markers (neutrophil count and platelet count). Unsurprisingly, there was a higher rate of diagnosis of localized infections with none of the neonates in the non-sepsis group having a diagnosis of localized infection (Table 4). Intraventricular haemorrage (IVH) was diagnosed in 43.6% (*n* = 48) of neonates, with *n* = 8 neonates having a grade 3 or greater IVH. A total of 11 neonates were diagnosed with Necrotising enterocolitis (NEC) (medical or surgical), with *n* = 5 in the culture-positive group and *n* = 6 in the culture-negative group. Only *n* = 1 neonate had surgically confirmed NEC and was in the culture-positive group.

**Figure 1:**
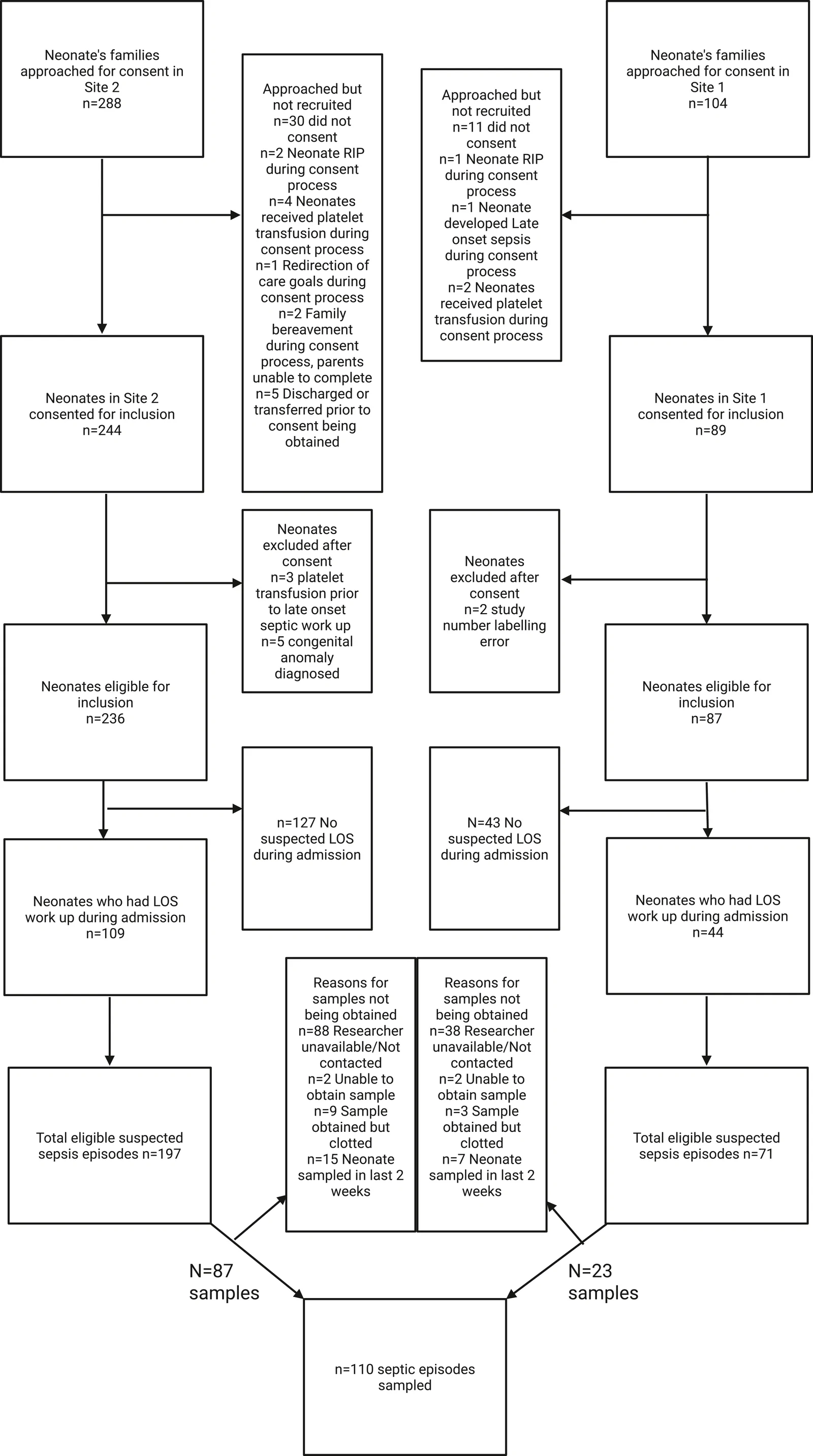
Flow diagram illustrating recruitment across both sites

**Table 1:** Demographic information regarding neonates who were investigated for sepsis during the study period

	Culture negative (*N* = 72)	Culture positive (*N* = 25)	Not sepsis (*N* = 171)	Overall (*N* = 268)
Gender				
Female	36 (50.0%)	13 (52.0%)	77 (45.0%)	126 (47.0%)
Male	36 (50.0%)	12 (48.0%)	94 (55.0%)	142 (53.0%)
Weight	880 [725–1,030]	700 [620–875]	940 [782–1,230]	910 [740–1,130]
Gestational age at birth	27.0 [25.3–28.0]	25.0 [23.7–25.9]	27.7 [26.1–29.3]	27.4 [25.4–28.9]
Maternal age	33.5 [30.0–37.0]	31.0 [29.0–34.0]	33.0 [29.0–37.0]	33.0 [29.0–37.0]
Ethnicity (maternal booking form)				
African	13 (18.1%)	2 (8.0%)	21 (12.3%)	36 (13.4%)
Asian	6 (8.3%)	2 (8.0%)	12 (7.0%)	20 (7.5%)
Black Irish	0 (0%)	0 (0%)	1 (0.6%)	1 (0.4%)
Brazilian	1 (1.4%)	0 (0%)	3 (1.8%)	4 (1.5%)
Irish Traveler	0 (0%)	1 (4.0%)	7 (4.1%)	8 (3.0%)
Other	0 (0%)	0 (0%)	0 (0%)	0 (0%)
White Irish	48 (66.7%)	18 (72.0%)	108 (63.2%)	174 (64.9%)
White Non-Irish	4 (5.6%)	2 (8.0%)	19 (11.1%)	25 (9.3%)
HP index	3.78 [−5.80–9.15]	3.14 [−3.15–8.94]	0.960 [−6.55–8.29]	1.63 [−6.50–8.47]
CRIB II	10.0 [8.00–12.0]	13.0 [11.0–14.0]	9.00 [7.00–12.0]	10.0 [7.00–12.0]
Maximum nSOFA score over first 48 h	0 [0–4.00]	4.00 [2.00–7.00]	0 [0–0]	0 [0–2.00]
Maximum VIS over first 48 h	0 [0–0]	0 [0–17.0]	0 [0–0]	0 [0–0]
Antenatal steroids				
None	5 (6.9%)	0 (0%)	21 (12.3%)	26 (9.7%)
Incomplete	7 (9.7%)	10 (40.0%)	25 (14.6%)	42 (15.7%)
Complete	60 (83.3%)	15 (60.0%)	125 (73.1%)	200 (74.6%)
Reason for septic work up				
Temperature instability	11 (15.3%)	2 (8.0%)	42 (24.6%)	55 (20.5%)
High CRP/IL-6/white cells	0 (0%)	0 (0%)	4 (2.3%)	4 (1.5%)
Thrombocytopenia	0 (0%)	2 (8.0%)	0 (0%)	2 (0.7%)
No clear indication	1 (1.4%)	0 (0%)	2 (1.2%)	3 (1.1%)
Candidal rash	1 (1.4%)	0 (0%)	0 (0%)	1 (0.4%)
Hypotension	1 (1.4%)	1 (4.0%)	0 (0%)	2 (0.7%)
Apnoeas/bradycardias/desaturations	16 (22.2%)	2 (8.0%)	64 (37.4%)	82 (30.6%)
Gastrointestinal symptoms	13 (18.1%)	5 (20.0%)	18 (10.5%)	36 (13.4%)
Increase in respiratory support	21 (29.2%)	7 (28.0%)	19 (11.1%)	47 (17.5%)
Skin redness/ulceration/abscess	8 (11.1%)	3 (12.0%)	10 (5.8%)	21 (7.8%)
Tachycardia	0 (0%)	1 (4.0%)	8 (4.7%)	9 (3.4%)
Poor appearance	0 (0%)	1 (4.0%)	3 (1.8%)	4 (1.5%)
Co-twin had septic work up	0 (0%)	0 (0%)	1 (0.6%)	1 (0.4%)
Abnormal gas	0 (0%)	1 (4.0%)	0 (0%)	1 (0.4%)
Recruited				
No sample obtained	36 (50.0%)	9 (36.0%)	113 (66.1%)	158 (59.0%)
Sample obtained	36 (50.0%)	16 (64.0%)	58 (33.9%)	110 (41.0%)
Leukocyte count (X10^9^/L)	16.1 [10.8–24.4]	16.7 [6.20–35.9]	13.5 [10.2–18.7]	14.3 [10.3–21.3]
Neutrophil count (X10^9^/L)	7.78 [4.03–14.9]	12.5 [3.84–25.2]	5.33 [3.60–10.2]	6.47 [3.66–12.7]
Platelet count (X10^9^/L)	305 [205–416]	167 [81.0–288]	336 [234–436]	309 [210–426]
CRP (mg/L)	1.00 [0–2.00]	3.50 [1.75–23.8]	0 [0–1.00]	0 [0–2.00]
Antenatal magnesium sulfate				
No MgSO_4_	14 (19.4%)	3 (12.0%)	28 (16.4%)	45 (16.8%)
MgSO_4_	58 (80.6%)	22 (88.0%)	143 (83.6%)	223 (83.2%)
Duration of mechanical ventilation (total for stay)	10.5 [2.00–21.0]	10.0 [6.00–18.0]	2.00 [0–8.00]	4.00 [1.00–12.3]
Duration on NIV for stay	29.5 [13.0–42.0]	12.0 [2.00–39.0]	32.0 [16.0–42.0]	31.0 [13.0–42.0]
Any bronchopulmonary dysplasia/chronic lung disease				
No BPD/CLD	8 (11.1%)	1 (4.0%)	26 (15.2%)	35 (13.1%)
Any BPD/CLD	55 (76.4%)	15 (60.0%)	141 (82.5%)	211 (78.7%)
Retinopathy of prematurity requiring treatment				
No ROP requiring treatment	54 (75.0%)	14 (56.0%)	148 (86.5%)	216 (80.6%)
ROP needing treatment	13 (18.1%)	4 (16.0%)	19 (11.1%)	36 (13.4%)
Death				
Alive at discharge	62 (86.1%)	16 (64.0%)	164 (95.9%)	242 (90.3%)
Dead prior to discharge	10 (13.9%)	9 (36.0%)	7 (4.1%)	26 (9.7%)

Categorical variables are displayed as *n* (%). Continuous variables are displayed as median (IQR). CRIB II, Clinical Risk Index for Babies II; VIS, vasomotor inotropic score; nSOFA, neonatal sequential organ failure assessment; VIS: vasomotor inotropic score; ROP, retinopathy of prematurity; BPD/CLD, bronchopulmonary dysplasia/chronic lung disease.

**Table 2:** Causative organisms identified in “culture-positive” sepsis group

Gram-positive organisms	Gram-negative organisms	Fungal organisms
Group B *Streptococcus* (GBS) *n* = 4	*Acinetobacter* spp. *n* = 1	*Candida albicans n =1*
	*Escherichia coli n* = 5	
*Bacillus cereus n* = 1	*Enterobacter cloacae n* = 1	
*Enterococcus faecium n* = 1^a^	*Klebsiella aerogenes n* = 1	
*Staphylococcus aureus n* = 2	*Klebsiella pneumoniae n* = 1	
*Staphylococcus capitis n* = 2	*Proteus mirabilis n* = 1	
*Staphylococcus epidermidis n* = 3	*Serratia liquefaciens n* = 1^a^	

a highlights organisms identified in a single blood culture from a single patient.

**Table 3:** Case definitions used for neonatal sepsis in the PARENT study

Culture positive	Culture negative	Non-sepsis
• Bacterial/fungal infection isolated from blood culture	• antibiotics continued for 5 or more days • planned continuation of 5 or more days or until death • clinical diagnosis of a localized infection or NEC without positive-blood culture • diagnosis of viral infection with antibiotics continued for 5 days	• antibiotics discontinued after 36–48 h or before 5 days • no suspicion of localized infection or NEC

**Table 4:** Comparison between sampled neonates included in the study, categorical variables compared using χ^2^ test, continuous variables compared using ANOVA, and ordinal variables (marked as ∗) compared using the Kruskal-Wallis test

	Culture negative (*N* = 36)	Culture positive (*N* = 16)	Not sepsis (*N* = 58)	*p* value
Gender				
Female	19 (52.8%)	6 (37.5%)	28 (48.3%)	0.596
Male	17 (47.2%)	10 (62.5%)	30 (51.7%)	–
Weight (grams)	962 [777.5–1,146.5]	862 [714.5–1,009.5]	1,010 [844.5–1,175.5]	0.31
Gestational age at birth (weeks)	27.3 [26–28.6]	25.7 [24.4–26.9]	27.3 [26.3–28.3]	0.04
Day of life when sepsis suspected	17.1 [8.5–25.5]	17.4 [9–25.2]	24.0 [14–34]	0.17
Maximum VIS over first 48 h∗	0 [0–0]	0 [0–12.5]	0 [0–0]	<0.001
Maximum nSOFA score over first 48 h∗	0.500 [0–4.00]	4.50 [1.50–7.25]	0 [0–0]	<0.001
Maximum level of respiratory support				
Room air	3 (8.3%)	0 (0%)	1 (1.7%)	NC
Low flow nasal cannula	0 (0%)	0 (0%)	3 (5.2%)	–
High flow nasal cannula	2 (5.6%)	1 (6.3%)	8 (13.8%)	–
Continuous positive airway pressure	8 (22.2%)	0 (0%)	24 (41.4%)	–
Bilevel positive airway pressure	2 (5.6%)	1 (6.3%)	12 (20.7%)	–
Volume guarantee ventilation	19 (52.8%)	11 (68.8%)	10 (17.2%)	–
Pressure control ventilation	2 (5.6%)	3 (18.8%)	0 (0%)	–
Reason for septic work up				
Temperature instability	5 (13.9%)	1 (6.3%)	15 (25.9%)	NC
High CRP/IL-6/white cells	0 (0%)	0 (0%)	2 (3.4%)	–
Thrombocytopenia	0 (0%)	2 (12.5%)	0 (0%)	–
No clear indication	1 (2.8%)	0 (0%)	1 (1.7%)	–
Candidal rash	1 (2.8%)	0 (0%)	0 (0%)	–
Apnoeas/bradycardias/desaturations	6 (16.7%)	1 (6.3%)	19 (32.8%)	–
Gastrointestinal symptoms	4 (11.1%)	4 (25.0%)	5 (8.6%)	–
Increase in respiratory support	13 (36.1%)	5 (31.3%)	7 (12.1%)	–
Skin redness/ulceration/abscess	6 (16.7%)	2 (12.5%)	4 (6.9%)	–
Tachycardia	0 (0%)	0 (0%)	3 (5.2%)	–
Poor appearance	0 (0%)	0 (0%)	2 (3.4%)	–
abnormal gas	0 (0%)	1 (6.3%)	0 (0%)	–
Leukocyte count (X10^9^/L)	16.3 [12.6–20]	22.3 [14–30.6]	16.1 [11.3–20.85]	0.08
Neutrophil Count (X10^9^/L)	8.82 [5.9–11.7]	16.2 [9.45–23]	8.62 [4.6–12.6]	0.006
Platelet count (X10^9^/L)	293 [214.5–371.5]	190 [118.5–261.5]	326 [244–408]	0.01
CRP (mg/L)	2.52 [0–5.16]	28.0 [5–51]	5.27 [0–13.07]	0.003
Pneumonia				
None	20 (55.6%)	14 (87.5%)	58 (100%)	<0.001
Pneumonia	16 (44.4%)	2 (12.5%)	0 (0%)	–
Cellulitis/soft tissue infection				
None	30 (83.3%)	14 (87.5%)	58 (100%)	0.00705
Soft tissue infection	6 (16.7%)	2 (12.5%)	0 (0%)	–
UTI				
None	36 (100%)	15 (93.8%)	58 (100%)	0.0516
UTI	0 (0%)	1 (6.3%)	0 (0%)	–
Death				
Alive at discharge	32 (88.9%)	11 (68.8%)	55 (94.8%)	0.0124
Dead prior to discharge	4 (11.1%)	5 (31.3%)	3 (5.2%)	–

NC, not calculable due to very small single cell counts; nSOFA, neonatal sequential organ failure assessment; VIS, vasomotor inotropic score; UTI, urinary tract infection.

### Both EP-enriched and EP-depleted plasma demonstrate proteomic differences between neonates with late-onset culture-positive sepsis and those with non-sepsis

EP-enriched neonatal plasma assessed by mass spectrometry showed both a statistical difference and a large fold-change difference in a small group of proteins in neonates diagnosed with culture-positive sepsis versus those with non-sepsis (culture-positive sepsis *n* = 11 and non-sepsis = 33, Figure 2A, full analysis attached as Data S1). Upregulated proteins comprised acute-phase reactants including serum amyloid A (SAA1, adjusted *p* value = 0.02, log_10_-fold change = 3.7/SAA2, adjusted *p* value = 0.009, log_10_-fold change = 5.18), liposaccharide-binding protein (LBP, adjusted *p* value = 0.03, log_10_-fold change = 1.6), and low-density lipoprotein receptor (LDLR, adjusted *p* value ≤ 0.0001, log_10_-fold change = 3.88); growth factors such as GLI pathogenesis related 2 (GLIPR2, adjusted *p* value = 0.0002, log_10_-fold change = 2.65) and brain associated soluble protein 1 (BASP1, adjusted *p* value = 0.004, log_10_-fold change = 1.5); as well as immune response associated proteins such as hematopoietic cell kinase (HCK, adjusted *p* value ≤ 0.0001, log_10_-fold change = 4.5), interleukin 1 receptor like 1 (IL1RL1, also known as suppression of tumorigenicity 2 (ST2), adjusted *p* value = 0.005, log_10_-fold change = 3.4), and interleukin 1 receptor antagonist (IL1RN, adjusted *p* value = 0.005, log_10_-fold change = 3.5). A single protein, tropomyosin 3 (TPM3, adjusted *p* value = 0.02, log_10_-fold change = −3.4), was downregulated to a statistically and fold-change significance level. Gene set enrichment analysis (GSEA) of Gene Ontology (GO) terms was performed and confirmed that proteins identified were associated with extracellular vesicle (EV) biology, inflammatory response, and immune responses (Figure 2B). To investigate if this was a unique EP proteomic profile and to assess the relevance of low-abundance proteins in plasma, an EP-depleted sample was assessed using Proximity extension analysis(PEA), which demonstrated large differences in a number of proteins relevant to cardiovascular disease (Figure 2C, full analysis included as Data S1). ST2/IL1RL1, a protein associated with cardiovascular dysfunction, was demonstrated in both EP-depleted and EP-enriched plasma. Other markers of cardiac dysfunction, such as N-terminal pro-brain natriuretic peptide (NT-proBNP, adjusted *p* value = 0.01, normalized protein expression (NPX) fold change = 2.3), and those of macrophage activation and acute inflammatory response, such as chitotriosidase-1 (CHIT1, adjusted *p* value = 0.0002, NPX fold change = 1.9), chitinase 3 like protein 1 (CHI3L1, adjusted *p* value ≤ 0.0001, NPX fold change = 1.6), and plasminogen activation inhibitor (PAI, adjusted *p* value = 0.0001, NPX fold change = 2.03) were also upregulated.

**Figure 2:**
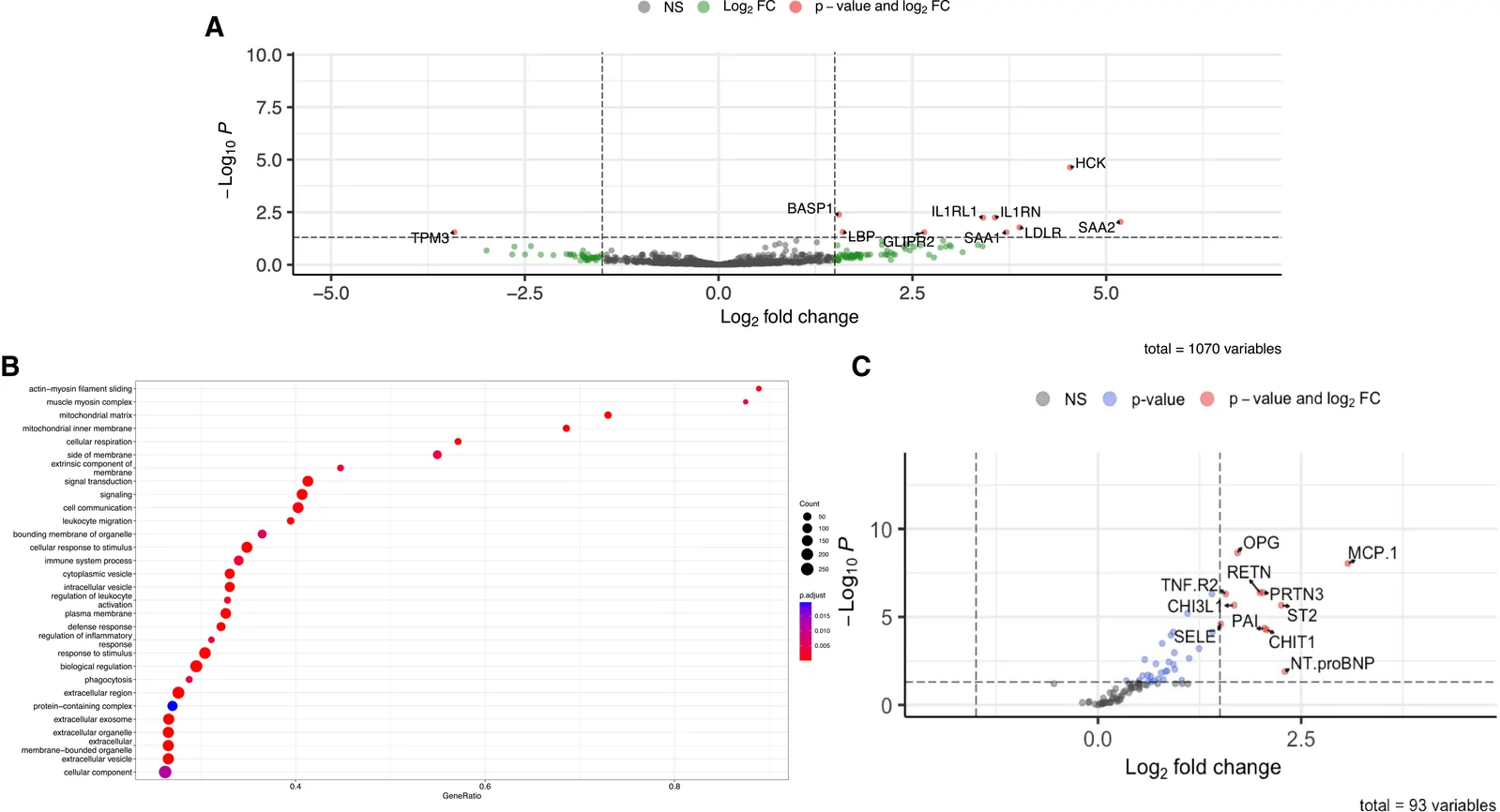
Diagram illustrating proteomic analysis of neonates with culture-proven sepsis versus those with non-sepsis (A) Volcano plot illustrating differences in protein expression detected on EV-enriched plasma by mass spectrometry in culture-proven sepsis versus non-sepsis, *x* axis displays log_10_-fold change, and *y* axis displays Benjamini-Hochberg adjusted *p* values (−log_10_p). (B) Dotplot demonstrating enriched terms per GSEA for differentially expressed proteins on mass spectrometry. (C) Volcano plot illustrating differences in protein expression detected on EV-depleted plasma by proximity extension assay in culture-proven versus non-sepsis, *x* axis displays NPX fold change, and *y* axis displays Benjamini-Hochberg adjusted *p* values (−log_10_p).

### Proteomic profiles between neonates without sepsis and with culture-negative sepsis do not differ in a statistically significant manner

Proteomic profiles in neonates with culture-negative sepsis were compared with those in neonates without a diagnosis of sepsis (Figures 3A and 3B). This demonstrated no statistically significant difference in protein expression between the two groups (see Data S1).

**Figure 3:**
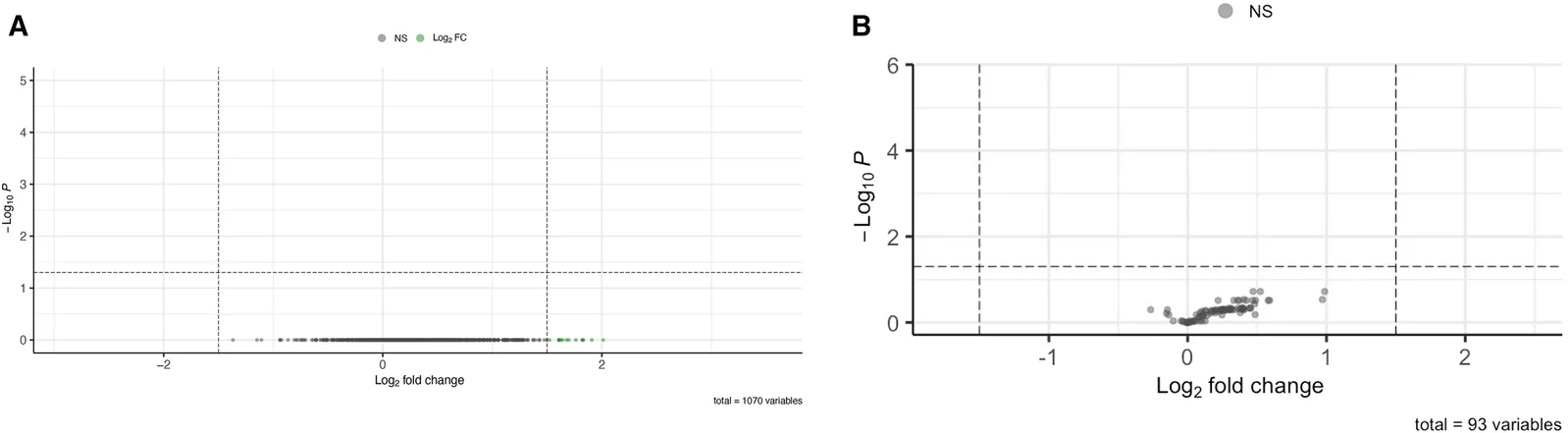
Diagram illustrating proteomic analysis of neonates with culture-negative sepsis versus those with non-sepsis (A) Volcano plot illustrating no statistically significant difference in protein expression detected on EV-enriched plasma by mass spectrometry in culture-negative versus non-sepsis. (B) Volcano plot illustrating no statistically significant difference in protein expression detected on EV-depleted plasma by proximity extension assay in culture-proven versus non-sepsis. *x* axis displays relative change in protein expression as either log_10_-fold change (Figure 3A) or normalized protein expression (NPX) fold change (B). *y* axis displays Benjamini-Hochberg adjusted *p* values (−log_10_p) (both A and B).

### EP profiles are similar between neonates with culture-proven sepsis, culture-negative sepsis, and non-sepsis

EP concentrations and putative cell of origin were assessed using flow cytometry. Given our a priori hypothesis, we selected flow cytometry markers associated with platelet and endothelial cell of origin. There was no statistically significant difference between culture-positive sepsis (*n* = 14), culture-negative sepsis (*n* = 29), and non-sepsis (*n* = 50) events in either EP concentration or the concentration of EPs stained with markers suggestive of endothelial or platelet origin (Figure 4).

**Figure 4:**
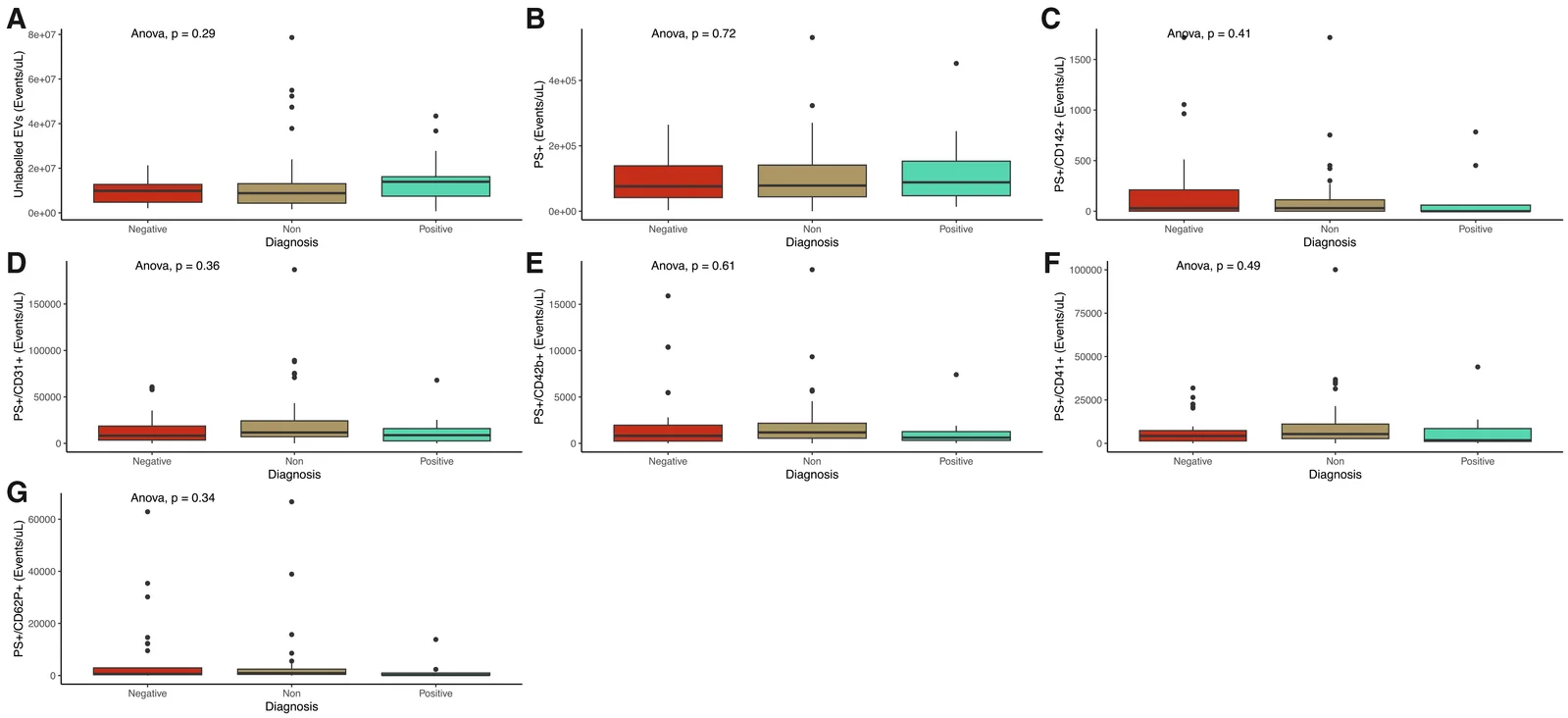
Boxplots describing both total and EV subpopulations across non-sepsis, culture-negative sepsis, and culture-proven sepsis (A) Unlabeled EVs, 200–1,000 nm; (B) phosphatidylserine (PS) single-positive EVs; (C) double-positive PS and tissue factor (TF)/CD142 EVs; (D) double-positive PS and platelet endothelial cell adhesion molecule (PECAM)/CD31 EVs; (E) double-positive PS and glycoprotein Ib alpha (GPIbα)/CD42 b EVs; (F) double-positive PS and alpha IIb integrin/CD41; and (G) double-positive PS and P-selectin/CD62P. Statistical comparisons were performed using ANOVA. All boxplots present the median with ±1.5 times interquartile range.

### A high ST2/IL1RL1 endotype of late-onset sepsis is associated with a high risk of death in this cohort

As a hypothesis-generating next step, differentially expressed proteins were examined via cluster analysis to determine if any “endotype” could be identified (Figure 5). On both proteomic datasets, a clear high-“ST2” endotype was identified on the heatmap and confirmed using model-based clustering methods in *n* = 8 neonates, with high rates of culture-positive sepsis (n = 6, 75% in EV-enriched mass spectrometry group, *n* = 7, 87.5% in EV-depleted group), worse severity of illness as assessed by VIS and nSOFA scores, and death (*n* = 3, 37.5% mortality in both groups) (Tables S1, S2, and S3).

**Figure 5:**
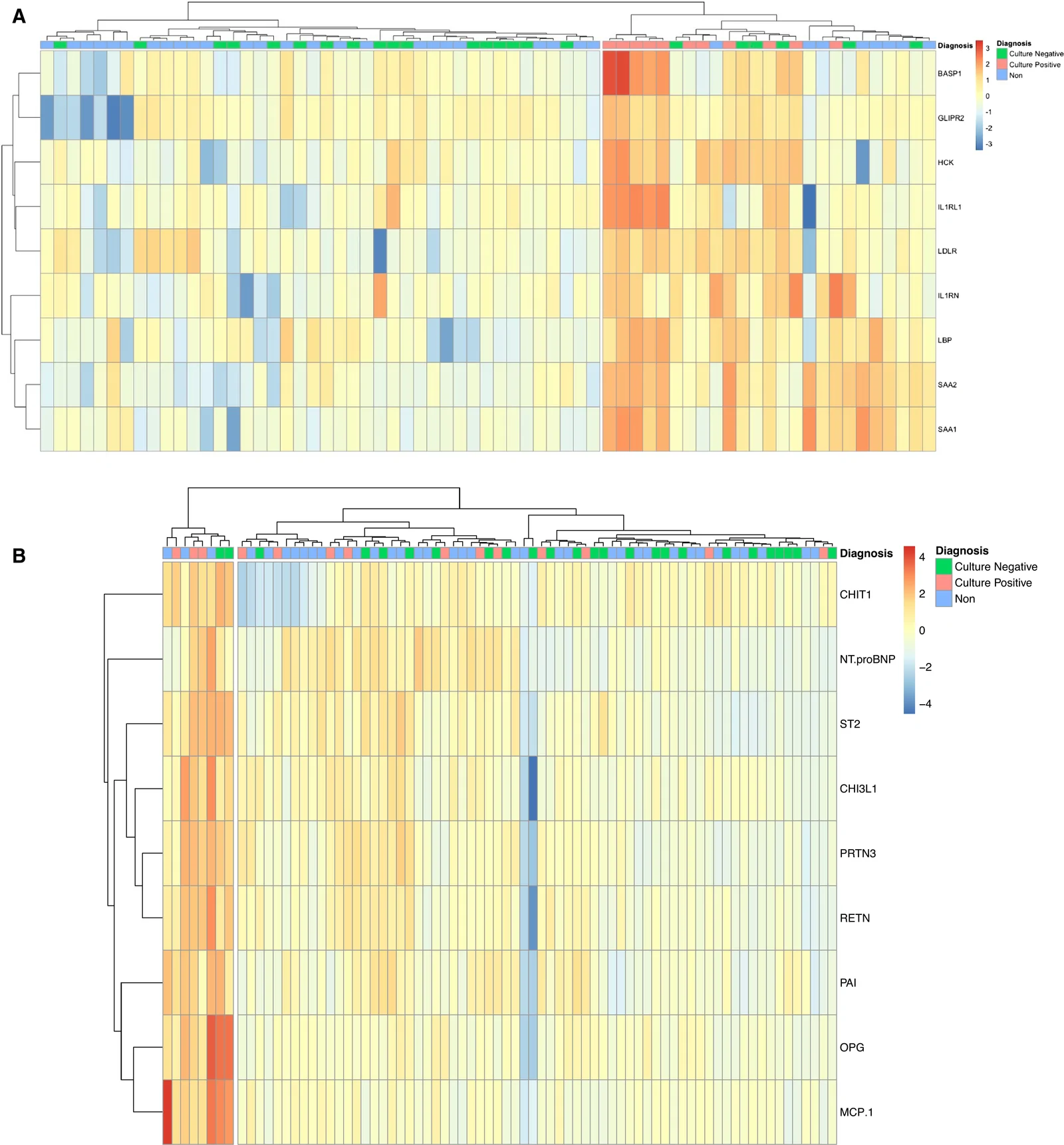
Heatmaps examining proteomic data Heatmaps demonstrating “high-risk” and “low-risk” clusters using (A) proteomic data from EV-enriched mass spectrometry and (B) proteomic data from EV-depleted PEA-based analysis.

## Discussion

The PARENT study was a prospective, multisite observational study that examined the role of platelet and endothelial activation in the identification of late-onset neonatal sepsis in neonates born at less than 34 weeks of cGA. To our knowledge, it is the largest, prospectively registered proteomic study performed to date in this population, with 110 sepsis episodes sampled and available for analysis in the cohort. A distinct circulating proteomic signature differentiated neonates with culture-positive late-onset sepsis from those clinically classified as non-sepsis. In contrast, culture-negative late-onset sepsis was indistinguishable from non-sepsis at both the proteomic and EV level. Model-based clustering identified “high-risk” clusters across both mass spectrometry and PEA, associated with death, culture positivity, and severity of illness. However, the small size of these clusters limited statistical analysis, and our findings should be considered hypothesis-generating.

Upregulation of many of the proteins in this cohort has been described elsewhere in the neonatal sepsis literature such as LBP, LDLR, and SAA1 and SAA2.^17^^,^^18^^,^^19^ Other protein upregulation patterns have not previously been described in the neonatal literature but are associated with adult sepsis phenotypes. High levels of soluble ST2/interleukin-1 receptor-like 1 (IL1RL1) have been associated with increased mortality in both adults admitted to ICU with sepsis and in mouse models, which was recapitulated in our cohort where it was also associated with high mortality and a high rate of culture-proven sepsis.^20^^,^^21^^,^^22^ Similarly, PAI has been well established as a predictor of all-cause mortality in other sepsis cohorts, even in the absence of overt disseminated intravascular coagulation.^23^ Similar findings have been demonstrated for other proteins upregulated in the culture-positive group such as osteoprotegerin (OPG) and monocyte chemoattractant protein 1 (MCP1).^24^^,^^25^

The lack of a proteomic signature or EV signature for culture-negative sepsis is also an important finding. It provides further evidence for the lack of biological underpinning for this diagnosis as currently defined in many neonatal studies.^9^^,^^11^^,^^26^ While it is important to note that many of these neonates are critically unwell, the broad definition of existing culture-negative sepsis diagnoses diminishes both the clinical and research utility of its continued use.^3^ Despite many years of debate, there remains no diagnostically validated means of identifying neonatal sepsis in the absence of microbiological proof from a sterile site.^4^^,^^27^ This has important implications for neonatal antimicrobial stewardship where this remains an important issue.^28^

The absence of statistically significant differences in total, endothelial, or platelet-derived EP concentrations between culture-positive groups and non-sepsis groups was unexpected. EPs have been implicated in adult sepsis as important mediators/indicators of disease.^29^^,^^30^^,^^31^ Although a recent study examining EVs in adults with sepsis showed limited differences in EV counts between survivors and non-survivors, it found profound differences in function, which were not measured in our cohort.^32^ Previously, our group demonstrated a sharp increase in EP concentrations on day of life 3 in preterm neonates who were sampled in the absence of concerns for sepsis.^33^ Given the high baseline concentrations circulating in this population, it may not be possible for further EP generation to be detected or generated in a sepsis cohort. Additionally, our study used a control group of neonates who were suspected of late-onset sepsis but received only a short course of antibiotics and were deemed clinically “non-sepsis.” This may reflect a higher baseline level of endothelial dysfunction within the cohort compared with a healthy control population due to non-sepsis co-morbidities, potentially obscuring detectable differences between the groups. The culture-positive group differed in a statistically significant way in gestational age at birth from the non-sepsis group and in hematological and clinical markers of inflammation across all groups. Importantly, this comparison reflects the clinically relevant distinction faced daily in practice, with the inherent heterogeneity of the identified patient cohorts under current definitions and the widespread of gestational ages admitted in a single NICU at any time. The small high-risk cluster identified using model-based and traditional clustering methods is intriguing given the recent publication of the ImmunoSep study in an adult cohort, identifying two separate sepsis phenotypes that responded to different therapeutic strategies.^34^ Further work to delineate sepsis endotypes in the neonatal population is both warranted and necessary to ensure neonatal patients receive the best care. Interestingly, neonates who were in the high-risk cluster on mass spectrometry of EV-enriched proteomics did not necessarily enter the high-risk cluster on EV-depleted PEA-based proteomics. This illustrates the potential of complex phenotypes within what is currently described clinically as “late-onset sepsis.”

In this prospective, multisite observational study, neonates ultimately diagnosed as having late-onset culture-positive sepsis differ proteomically from those who were diagnosed as not septic. This was driven by a high ST2/IL1RL1 endotype in this cohort described by both mass spectrometry and PEA and not previously described in a neonatal population. Neonates with culture-negative late-onset sepsis did not differ in a statistically significant manner from those with non-sepsis. EV concentrations were similar across all three groups, which may represent high baseline production of EVs in this cohort. Further work is required to validate these findings and apply them clinically to identify neonates who require aggressive sepsis management strategies from those who do not.

### Limitations of the study

There are a number of limitations to this study. Not every neonate who had been consented for inclusion provided a sample. It is important to note that these neonates did not appear to differ significantly from those who were included (Tables S1–S3), but whether other subtle differences exist within the cohort is difficult to say. The number of neonates who were both sampled and had culture-proven sepsis was low (*n* = 16), meaning that further validation of the novel markers described in this cohort is required, or subtle changes in EP concentration/phenotype may have been obscured. Given the very small sample volumes (0.5–1.3 mL), not every neonate had every investigation performed, which limits complete comparability between PEA, mass spectrometry, and flow cytometric analysis. Similarly, the small sample volumes precluded full characterization of EPs in accordance with minimal information for studies of EV (MISEV) guidance.^35^

## Resource availability

### Lead contact

Requests for further information and resources should be directed to and will be fulfilled by the lead contact, Daniel O’Reilly (danieloreilly@alumnircsi.com).

### Materials availability

This study did not generate new unique reagents.

### Data and code availability

• Associated proteomic datasets are available on the Proteomics Identifications Database (PRIDE, accession number: PXD066068 [mass spectrometry], PAD000028 [PEA]). Data regarding extracellular vesicles and used to prepare boxplots is included in the accompanying Data S1. Deidentified human data are available on reasonable request and within the limitations of general data protection regulations.
• This paper does not report original code.
• Any additional information required to reanalyze the data reported in this paper is available from the lead contact upon request

## Acknowledgments

P.B.M. has received funding from Science Foundation Ireland AI for Good Challenge Fund (19/FIP/AI/7490R2) and a Disruptive Technology Innovation Fund DT 2021 0373A from the Department of Enterprise, Trade and Employment, Ireland. D.O’.R. has been awarded the Irish Clinical Academic Training (ICAT) Program and supported by the Wellcome Trust and the Health Research Board (grant no. 203930/B/16/Z) and the Health Service Executive, National Doctors Training and Planning, and the Health and Social Care, Research and Development Division, Northern Ireland. The authors would also like to gratefully acknowledge the participants and their parents/guardians who took part from both the Rotunda Hospital and National Maternity Hospital for their shared commitment to advancing neonatal care.

## Author contributions

D.O’.R., N.M., and P.B.M. conceptualized the study. D.O’.R., L.M., L.R., C.A.M., and C.M.M. recruited and consented patients/their guardians. Initial sample processing was performed by D.O’.R. and L.M. D.O’.R., E.F., K.W., and R.I. performed the laboratory analysis. D.O’.R. and I.C.G. drafted statistical analysis plan and carried out statistical analysis. D.O’.R. collected clinical data and managed data on site. J.O’.L. provided logistical laboratory support and sample management. F.N.A., R.J.D., P.D., C.N.M., A.C., N.M., and P.B.M. provided supervisory support and subspeciality expertise. D.O’.R. drafted the initial manuscript. Manuscript was reviewed and further edited by all contributing authors prior to submission.

## Declaration of interests

The authors declare no competing interests.

## STAR★Methods

### Key resources table

**Table:** 

REAGENT or RESOURCE	SOURCE	IDENTIFIER
**Antibodies**		

Annexin V FITC	Biolegend	AB_2561291 (BioLegend Cat. No. 640905)
CD41 PB	Biolegend	AB_10681721 (BioLegend Cat. No. 303713)
CD42B-APC	Biolegend	AB_2113770 (BioLegend Cat. No. 303912)
CD62P-PE	Biolegend	AB_314477 (BioLegend Cat. No. 304905)
CD31-PB	Biolegend	AB_1877196 (BioLegend Cat. No. 303113)
CD142-APC	Biolegend	AB_2564567 (BioLegend Cat. No. 365205)

**Biological samples**		

Preterm neonatal samples collected specifically for study	Rotunda Hospital/National Maternity Hospital	NA

**Chemicals, peptides, and recombinant proteins**		

Annexin V Binding Buffer	BD Pharmigen	Cat no: 556454

**Critical commercial assays**		

Olink Explore Cardiovascular III Panel	Olink Bioscience	SCR_003899

**Deposited data**		

Mass spectrometry data	PRIDE database	PXD066068
Proximity Extension Analysis data	PRIDE database	PAD000028

**Software and algorithms**		

R Statistical Software	R Project for Statistical Computing	SCR_001905
Perseus	Max Planck Institute	SCR_015753
Kaluza	Beckman Coulter	SCR_016182
Impute LCMD	Comprehensive R archive network	https://doi.org/10.32614/CRAN.package.imputeLCMD
Linear models for microarray and -omics analysis (LIMMA)	http://bioinf.wehi.edu.au/limma/	SCR_010943
EnhancedVolcano	https://bioconductor.org/packages/EnhancedVolcano/	SCR_018931
Pheatmap	https://www.rdocumentation.org/packages/pheatmap/versions/0.2/topics/pheatmap	SCR_016418
mclust	Comprehensive R Archive Network	SCR_027153
clusterprofiler	http://bioconductor.org/packages/release/bioc/html/clusterProfiler.html	SCR_016884

**Other**		

Clinical trial registration	https://clinicaltrials.gov/study/NCT05530330	NCT05530330

### Experimental model and study participant details

#### Study participants

Neonates recruited for the PARENT study were recruited from both the Rotunda Hospital and National Maternity Hospital between August 2022-Feburary 2025. Neonates born between 23 + 0 and 33+6 weeks cGA were eligible for inclusion. Exclusion criteria included being diagnosed with a major congenital anomaly or condition likely to affect the bone marrow or if the neonate received a platelet transfusion prior to sampling. Neonates and their families were offered the chance to participate when they were born, If informed consent was granted (*n* = 323). Where these infants subsequently had investigations for potential late onset sepsis (LOS), defined as occurring beyond 72 h of life and occurring prior to when their eventual diagnosis was determined, an additional sample (0.5–1.3 mL citrated plasma) was requested (*n* = 268, *n* = 110 events successfully sampled). Following sampling, clinical care continued as per treating clinician discretion. Neonates were later classified based on blood culture positivity and duration of treatment, as either “culture positive” sepsis (*n* = 16), where a neonate had blood culture proven bacteraemia/fungaemia and were treated with at least 5 days of antibiotics/antimicrobials for same, “culture negative” sepsis (*n* = 36) where a neonate did not have a proven bacteraemia/fungaemia but received at least 5 days of antibiotics or “non sepsis” (*n* = 58), where a neonate was initially investigated for sepsis but subsequently had less than 5 days of antibiotics (Table 4). This predefined, preregistered definition was utilised as it is in keeping with recent literature examining neonatal sepsis given the lack of validated diagnostic criteria in this population.^9^^,^^11^^,^^28^ Research ethics committees on both sites approved the study (NMH REC EC24.2022/Rotunda hospital REC-2022-0088). This study is reported according to the Strengthening the Reporting of Observational Studies in Epidemiology for Newborn Infection (STROBE-NI) guidelines.^36^ Sex of neonates was recorded and included as a variable for analysis but did not differ significantly between groups.

Bronchopulmonary dysplasia/chronic lung disease was defined by the Neonatal Research Network (NRN) definition of requirement for oxygen for 28 days or greater (8). Necrotising enterocolitis (NEC) diagnosis was defined by intention to treat for 5 days or more medically (medical NEC) or the requirement for surgery (surgical NEC). Intraventricular haemorrhage (IVH) was reported by paediatric radiologists on each site. Decision to treat retinopathy of prematurity (ROP) was made on both sites as part of an ROP screening program.

### Method details

#### Preparation of platelet poor plasma

All sodium citrate plasma samples were processed as soon as possible after collection and within 6-8 hours of sampling to allow for collection of “discard” plasma from clinically processed coagulation tests, the majority of samples were collected within 30 minutes to an hour of sampling. This was allowed on study protocol to prevent excessive blood sampling from each individual neonate as per best practice for research in neonates.^37^ Each sample was visually inspected for evidence of clot formation and discarded if found. Platelet Poor Plasma (PPP) was generated by double centrifugation at 1187 xg for 6 minutes at room temperature with full brake applied. Centrifuged plasma was removed from the initial tube without disturbing the formed pellet in between centrifugation steps to minimise platelet contamination. Platelet counts were not confirmed post centrifugation but the method employed was consistent with other studies which reliably produced PPP and which have been previously utilised to produce PPP in this cohort.^33^^,^^38^ PPP was aliquoted into vials and stored between -70 to -80°C until analysis.

#### Preparation of neonatal EP enriched plasma using benchtop centrifugation

EPs were enriched from 50ul PPP by centrifugation at 20,800xg at 10°C for 10 minutes in an Eppendorf 5810R centrifuge with an SL-140 rotor. The supernatant was removed, and EP pellets were resuspended in 1mL filtered PBS, vortexed and spun again as before. Presence of EPs in the enriched plasma was established using flow cytometry as described below as well as assessment of expression of extracellular vesicle specific markers on mass spectrometry (supporting data values file).

#### Flow cytometry of neonatal EVs in enriched plasma

The supernatant was removed and washed EP pellets were resuspended in 230uL 1X Annexin V Binding Buffer (BD Pharmingen). 50μl of this EP suspension was separated into 4 tubes (unstained, annexin-V only, platelet markers-CD41, CD42b, CD62P, annexin-V and endothelial markers- CD31, CD142, annexin-V) and stained at room temperature for 20 minutes in the dark with corresponding fluorophore conjugated antibodies; Annexin-V-FITC, CD41-PB, CD42b-APC, CD62P-PE, CD31-PB, CD142-APC, (2.5 μl per antibody per test; BioLegend, San Diego, USA). Following incubation binding was quenched by addition of 300μl Annexin-V binding buffer, and centrifuged at 20,800xg, 10 minutes at 10°C. The supernatant was removed and stained EPs resuspended in 200μL Annexin-V binding buffer and loaded onto a 96 well plate for acquisition on a Cytoflex S flow cytometer (Beckman Coulter, USA). Samples were acquired at 10μL/min and recorded for 150 seconds. A buffer only sample (0.22 μm filtered PBS) was assayed in between patient samples using the same settings to ensure sufficiently low background between samples (<50 events/second). Gating was used to exclude any particles which may have represented residual platelets. Calibration beads were run weekly to apply appropriate size gates. Output .fcs files were gated and batched analysed using Kaluza Analysis software (Beckman Coulter v_2.2).

#### Preparation of EP enriched pellets for proteomic analysis

EP enriched pellets were prepared as described above. Urea lysis was performed to denature proteins and prepare EP samples for downstream on-bead digestion and proteomic analysis In brief, to each 20 μg EP sample, 40 μL of urea buffer (8 M urea, 50 mM Tris-HCl, pH ∼8.0) was added, followed by brief vortexing and sonication (level 10, 10 s, probe sonicator). Samples were reduced with 4.8 μL of 100 mM DTT (final ∼8 mM) at 37 °C, 1000 rpm for 30 min. For alkylation, 6.5 μL of 200 mM IAA (final ∼20 mM) was added, and samples were incubated in the dark at 37 °C, 1000 rpm for 30 min on a ThermoMixer (Eppendorf).

Proteins were digested using on bead protein precipitation using the Kingfisher flex (Thermo Fisher Scientific). For proteolytic digestion 20 μg of protein was digested in a 1:20 Trypsin/Lys-C enzyme:substrate ratio at 37 °C overnight. To terminate enzymatic activity, formic acid was added to a final concentration of 2%. Purification and sample loading was carried out using Evotips (Evosep Biosystems, Odense, Denmark). Each Evotip was activated using ethanol and centrifuged at 1000 ×g for 60 seconds. The tips were then equilibrated with 20 μL of 0.2% formic acid and centrifuged again at 1000 ×g for 60 seconds. Tryptic peptides were (20 μL) were loaded onto equilibrated Evotips in a random order with respect to clinical group and centrifuged at 700 ×g for 120 seconds. To maintain hydration of the tips prior to LC-MS injection, 200 μL of 0.2% formic acid was added to each tip and centrifuged briefly at 700 ×g for 10 seconds. For large batch processing, solvent levels were monitored and replenished throughout the run to prevent drying out of any Evotips.

#### Mass spectrometry on neonatal EP enriched plasma using data independent acquisition by neural networks

Samples were run on a Bruker timsTof Pro (Bruker, USA) mass spectrometer connected to an Evosep One liquid chromatography system (Evosep, Denmark). Tryptic peptides were resuspended in 0.1% trifluoroacetic acid and each sample was loaded on to an Evosep tip (Evosep, Denmark). The Evosep tips were placed in position on the Evosep One, in a 96-tip box. The autosampler is configured to pick up each tip, elute and separate the peptides using a set chromatography method (30 samples a day).^39^ The mass spectrometer was operated in positive ion mode with a capillary voltage of 1500 V, dry gas flow of 3 l/min and a dry temperature of 180 °C. All data was acquired with the instrument operating in a data dependent analysis parallel accumulation serial fragmentation mode (dda-PASEF). Trapped ions were selected for MS/MS using parallel accumulation serial fragmentation (PASEF). A scan range of (100-1700 m/z) was performed at a rate of 5 PASEF MS/MS frames to 1 MS scan with a cycle time of 1.03s.^40^

Prior to creating a data independent analysis (DIA) method, a pool of the combined trypsin digested samples was analysed using dda-PASEF method described above. The resultant file was used to create the dia-PASEF method within Bruker timsControl software. The scan mode “dia-PASEF” was selected and the pooled sample dda-PASEF file was opened in the window editor in the MS/MS tab. Once opened the adjustable rhomboid was used to select the area of the heat map where the identifiable peptides (central region in the heat map containing peptides with charge states from +2 to +5) can be found. All data was acquired using data independent analysis parallel accumulation serial fragmentation (dia-PASEF). Dia-PASEF settings used were mass steps per cycle 31, mobility overlap 0.00, mass overlap 1.00 Da, mass range 324.8 – 1100.8 m/z, cycle time estimate 1.80 seconds.^41^

Samples were run in a single analytic run to avoid batch effects, with pooled reference samples (*n* = 4) interspersed through the analytical run to examine for drift using Coefficient of Variation which was stable throughout (Cv=4.5-5 on raw label free quantification data) similar to previously published methods using DIA.^42^ Each sample was run as a single technical replicate with biological replicates as reported below.

Data acquired using dia-PASEF was analysed using DIA-NN 2.1.0 (Data-Independent Acquisition by Neural Networks).^43^ The Homo sapiens subset from the Uniprot Swissprot database (reviewed) was used to generate a spectral Library within DIA-NN (library free mode). Specific search settings included cysteine carbamidomethylation enabled as a fixed modification, methionine oxidation and n-terminal acetylation as variable modifications, maximum missed cleavages 1, the machine learning setting was set to neural networks cross validated, cross run normalisation was set to retention time dependent and library generation was set to identifications, retention time & ion mobility profiling. All output was filtered at 0.01 FDR.

#### Proximity Extension Analysis of EP depleted plasma

Proximity Extension Analysis was performed on 96 low abundance proteins associated with cardiovascular dysfunction (Olink Target 96 Cardiovascular III). The supernatant generated on first centrifugation of platelet poor plasma for EP enrichment was collected and analysed using Olink in University College Dublin Clinical Research Centre (UCD CRC).

### Quantification and statistical analysis

Statistical analysis on EP proteomic contents was performed as follows and as described previously.^44^ Initial statistical analysis including significance testing was performed using Perseus software (Max Planck institute of biochemistry, Bayern, Germany).^45^ Only proteins identified in at least 50% of the replicate runs in at least one timepoint or population were included. Differences in protein expression were determined using an unpaired t-test with an FDR of 5% and a minimal fold change of 0.1 within Perseus software. All further statistical analyses were performed using the R statistical software.^46^^,^^47^ Mass spectrometry derived proteomic data set was imputed using quantile regression imputation for left censored data (QRILC).^48^ Mass spectrometry data was normalised using Log10 transformation.

EP depleted proteomic analysis performed using PEA, was put through the same statistical pipeline as described below without imputation or initial significance testing. PEA was reported as Normalised Protein Expression (NPX) and did not require normalisation.

Differential expression analysis was performed using the “Linear models for microarray and -omics analysis” (LIMMA) package with *p*-values adjusted for multiple pairwise hypothesis testing using the Benjamini-Hochberg method.^49^

Volcano plots were generated using “EnhancedVolcano”.^50^ The ‘Pheatmap’ package was used to generate heatmaps, clustering was performed using Euclidean distance for proteins and Ward’s criterion (WardD2) for patients.^51^ Gaussian mixture model clustering and probabilistic cluster membership analysis was performed using the “mclust” package.^52^ Gene set enrichment analysis (GSEA) was performed using the package “clusterprofiler” comparing against the Gene Ontogeny (GO) database.^53^^,^^54^^,^^55^

Comparison of clinical groups was completed using ANOVA for continuous data, Kruskal Wallis for ordinal data and Χ^2^ for categorical data. Where ANOVA demonstrated a statistically significant difference, a post hoc Tukey’s Honest significant difference test was applied. For all analysis *n* = number of samples, *p* < 0.05 was considered statistically significant throughout the manuscript.

## Supplemental information

Document S1. Tables S1–S3

Data S1. Full results of proteomic and EV analysis
