# Supplementary material for: Proteomic and extracellular particle changes in preterm neonates with suspected late-onset sepsis
Source: iScience. 2026 Oct 15;29(11):117895. doi: 10.1016/j.isci.2026.117895 (PMC13691268; doi:10.1016/j.isci.2026.117895)
Supplement: Supplementary file 1 [file mmc1.pdf]

iScience, Volume *29*

Supplemental information

Proteomic and extracellular particle changes in preterm neonates with suspected late-onset sepsis

Daniel O'Reilly, Lauren Murphy, Ella Fouhy, Laura Ryan, Claire Anne Murphy, John O'Loughlin, Fionnuala Ní Áinle, Isobel Claire Gormley, Kieran Wynne, Rosanna Inzitari, Peter Doran, Richard J. Drew, Craig N. Morrell, Carmel Maria Moore, Anna Curley, Naomi McCallion, and Patricia B. Maguire

	Site 1 (N=87)	Site 2 (N=236)	Overall (N=323)
**Gender**			
Female	47 (54.0%)	102 (43.2%)	149 (46.1%)
Male	40 (46.0%)	134 (56.8%)	174 (53.9%)
**Weight (grammes)**	1180[868-1490]	1420[998-1660]	1380[940-1630]
**Gestational age at birth (weeks)**	29.1[27.3-30.8]	30.4[27.7-32.3]	30.0[27.6-32.0]
**Maternal age (years)**	33.0[30.0-37.0]	33.0[30.0-37.0]	33.0[30.0-37.0]
**CRIB II**	6.00[4.00-11.0]	5.00[2.00-9.00]	6.00[3.00-10.0]
**Ethnicity (Maternal Booking form)**			
African	7 (8.0%)	16 (6.8%)	23 (7.1%)
Asian	3 (3.4%)	18 (7.6%)	21 (6.5%)
Black Irish	0 (0%)	1 (0.4%)	1 (0.3%)
Brazilian	2 (2.3%)	0 (0%)	2 (0.6%)
Irish Traveller	1 (1.1%)	4 (1.7%)	5 (1.5%)
Other	0 (0%)	2 (0.8%)	2 (0.6%)
White Irish	69 (79.3%)	162 (68.6%)	231 (71.5%)
White Non-Irish	5 (5.7%)	33 (14.0%)	38 (11.8%)
**HP index**	-0.350[-6.16-6.37]	3.07[-6.53-10.1]	2.12[-6.54-8.94]
**Antenatal Steroids**			
None	6 (6.9%)	21 (8.9%)	27 (8.4%)
Incomplete	7 (8.0%)	37 (15.7%)	44 (13.6%)
Complete	74 (85.1%)	178 (75.4%)	252 (78.0%)
**Antenatal Magnesium Sulphate**			
No MgSO4	20 (23.0%)	92 (39.0%)	112 (34.7%)

	Site 1 (N=87)	Site 2 (N=236)	Overall (N=323)
MgSO4	67 (77.0%)	144 (61.0%)	211 (65.3%)
**Suspected Late onset sepsis during admission**			
No suspected LOS	43 (49.4%)	127 (53.8%)	170 (52.6%)
Suspected LOS	44 (50.6%)	109 (46.2%)	153 (47.4%)
**Total duration of mechanical ventilation in days**	1.00[0-5.50]	0[0-2.00]	0[0-3.00]
**Total duration on NIV for stay in days**	15.0[5.00-41.0]	6.00[2.00-19.0]	8.00[2.00-27.5]
**Any Bronchopulmonary dysplasia/Chronic Lung Disease**			
No BPD/CLD	37 (42.5%)	135 (57.2%)	172 (53.3%)
Any BPD/CLD	45 (51.7%)	84 (35.6%)	129 (39.9%)
**Retinopathy of Prematurity requiring treatment**			
No ROP requiring treatment	81 (93.1%)	215 (91.1%)	296 (91.6%)
ROP needing treatment	1 (1.1%)	13 (5.5%)	14 (4.3%)
**Death**			
Alive at discharge	80 (92.0%)	226 (95.8%)	306 (94.7%)
Dead prior to discharge	7 (8.0%)	10 (4.2%)	17 (5.3%)

Supplemental Table 1: Demographic information regarding neonates across both recruiting sites, categorical variables displayed as n(%), continuous variables displayed as median (Interquartile range). HP Index= Haase-Pratschke (HP) index, a measure of area level socioeconomic deprivation.

	High IL1RL1/ST2 (EV mass spectrometry, n=8)	Low IL1RL1/ST2 (EV mass Spectrometry, n=59)	Total (EV mass Spectrometry, n=67)	High IL1RL1/ST2 (EV depleted PEA, n=8)	Low IL1RL1/ST2 (EV depleted PEA, n=67)	Total (EV depleted PEA, n=75)
**Gender (n ,% male)**	8 (100%)	26 (44.1%)	34 (50.7%)	4(50%)	34 (50.7%)	38 (50.7%)
**Birth Weight (g, IQR)**	783 (710-840)	980 (753-1220)	940 (735-1130)	768 (633-849)	940 (730-1130)	930(698-1110)
**Gestational age at birth (weeks, IQR)**	24.5 (24-25.1)	27.7 (26.1-29.8)	27.4 (25.6-29.3)	24.3 (23.9-25.9	27 (25.4-29.1)	26.7 (25.1-28.9)
**CRIB II**	14(13.3-14)	8(5.5-11)	9 (7-12)	12.5 (10.5-14)	10 (7-12.8)	10 (7-13)
**Day of life when sepsis investigated**	13 (7.25-39)	10(7-26)	11 (7-27.5)	13 (7.8-33.5)	13 (7-25)	13 (7-25)
**Maximum nSOFA**	3 (0-7)	0 (0-2)	0 (0-2)	5.5 (4.25-11)	0 (0-2)	0 (0-3.5)
**Maximum VIS**	0 (0-5)	0	0	5 (0-24)	0	0
**Antenatal Steroids**<br>- Complete<br>- Partial<br>- None	 7(87.5%)<br>1 (12.5%)<br>0	 50(84.5%)<br>8(13.6%)<br>6 (10.2%)	 52 (77.6%)<br>9 (13.4%)<br>6 (9%)	 6 (75%)<br>2(25%)<br>0	 46(68.7%)<br>15 (22.4%)<br>6 (9%)	 52(69.3%)<br>17(22.7%)<br>6 (8%)
**MGSO4 antenatally (n, %)**	7 (87.5%)	50 (84.7%)	57 (85.1%)	8 (100%)	56 (83.6%)	64 (85.3%)
**Duration of mechanical ventilation (days, IQR)**	15.5 (8.5-22)	4 (1-12)	6 (2-17)	10.5 (8.5-16.8)	5 (1-20.5)	6 (2-20.5)
**Duration of non-invasive ventilation (days, IQR)**	27 (7-39.8)	4 (1-12)	6(2-17)	27.5 (6-39.8)	31 (13.5-40)	30 (10.5-40)

**ROP requiring treatment (n, %)**	2 (25%)	9(15.3%)	11(16.4%)	0	15 (22.4%)	15 (20%)
**Blood culture positive (n (%))**	6(75%)	6 (10.2%)	12 (17.9%)	7 (87.5%)	6 (9%)	13 (17.3%)
**Organism on blood culture**	*1=Candida albicans 1=Enterococcus faecium/Serratia liquifaciens 2=Group B streptococcus 1=Klebsiella aerogenes 1=Proteus mirabilis*	*2=Staphlococcus epidermidis 1=Group B Streptococcus 2=E.coli 1=Enterobacter cloacae*	*2=Staphlococcus epidermidis 1=Group B Streptococcus 2=E.coli 1=Enterobacter cloacae 1=Candida albicans 1=Enterococcus faecium/Serratia liquifaciens 2=Group B streptococcus 1=Klebsiella aerogenes 1=Proteus mirabilis*	*3=Group B Streptococcus 2=E. col 1=Klebsiella aerogenes 1=Proteus mirabilis*	*1=E.coli 1=Enterococcus faecium/Serratia liquifaciens 1=Enterobacter cloacae 1=Staphylococcus capitis 1=Staphylococcus epidermidis 1=Staph aureus*	*3=E.coli 3=Group B Strep 1=Klebsiella aerogenes 1=Proteus mirabilis 1=Enterococcus faecium/Serratia liquifaciens 1=Enterobacter cloacae 1=Staphylcoccus capitis 1=Staphylococcus epidermidis 1=Staphylococcus aureus*
**Viral organism identified**	1 (12.5%)	2 (3.3%)	3 (4.4%)	0	2 (2.89%)	2(2.6%)
**Localised infection**						
- Meningitis	0 (0%)	0 (0%)	0 (0%)	0 (0%)	0 (0%)	0 (0%)
- Pneumonia	1 (12.5%)	11 (18.6%)	12(17.9%)	1 (12.5%)	11 (15.9%)	12 (16%)
- Cellulitis	0 (0%)	4 (6.8%)	4 (5.9%)	0 (0%)	7(10.4%)	7 (9.3%)
	0 (0%)	0 (0%)	0 (0%)	0 (0%)	1 (1.4%)	1 (1.3%)

- Urinary tract infection						
**Necrotising enterocolitis**	2 (25%)	6 (10.1%)	8 (11.9%)	3 (37.5%)	6 (8.7%)	9 (12%)
**Death prior to discharge (n, (%))**	3 (37.5%)	4 (6.8%)	7 (10.4%)	3 (37.5%)	5 (7.5%)	8 (10.7%)

Supplemental table 2: Comparison between demographics and clinical diagnosis and outcomes between high IL1RL1/ST2 cluster and non-high IL1RL1/ST2 cluster by both proteomic methodologies as determined by model based clustering.

	No sample obtained (N=158)	Sample obtained (N=110)	Overall (N=268)
**Gender**			
Female	73 (46.2%)	53 (48.2%)	126 (47.0%)
Male	85 (53.8%)	57 (51.8%)	142 (53.0%)
**Weight (grammes)**	915[750-1130]	900[730-1110]	910[740-1130]
**Gestational age at birth (weeks)**	27.4[25.7-28.3]	27.0[25.3-29.1]	27.4[25.4-28.9]
**Maternal age (years)**	33.0[29.0-37.0]	33.0[29.0-36.3]	33.0[29.0-37.0]
**CRIB II (score)**	10.0[8.00-12.0]	10.0[7.00-13.0]	10.0[7.00-12.0]
**Maximum nSOFA score over first 48 hours**	0[0-1]	0[0-2]	0[0-2]
**Maximum VIS over first 48 hours**	0[0-0]	0[0-0]	0[0-0]
**Antenatal Steroids**			
None	13 (8.2%)	13 (11.8%)	26 (9.7%)
Incomplete	22 (13.9%)	20 (18.2%)	42 (15.7%)
Complete	123 (77.8%)	77 (70.0%)	200 (74.6%)
**Antenatal Magnesium Sulphate**			
No MgSO4	25 (15.8%)	20 (18.2%)	45 (16.8%)
MgSO4	133 (84.2%)	90 (81.8%)	223 (83.2%)

	No sample obtained (N=158)	Sample obtained (N=110)	Overall (N=268)
**Total Duration of mechanical ventilation in days**	3.00[0.625-11.8]	6.00[1.00-17.3]	4.00[1.00-12.3]
**Total Duration on NIV for stay in days**	31.0[13.0-42.0]	29.0[10.8-40.0]	31.0[13.0-42.0]
**Any Bronchopulmonary dysplasia/Chronic Lung Disease**			
No BPD/CLD	20 (12.7%)	15 (13.6%)	35 (13.1%)
Any BPD/CLD	126 (79.7%)	85 (77.3%)	211 (78.7%)
**Retinopathy of Prematurity requiring treatment**			
No ROP requiring treatment	131 (82.9%)	85 (77.3%)	216 (80.6%)
ROP needing treatment	18 (11.4%)	18 (16.4%)	36 (13.4%)
**Death**			
Alive at discharge	144 (91.1%)	98 (89.1%)	242 (90.3%)
Dead prior to discharge	14 (8.9%)	12 (10.9%)	26 (9.7%)

Supplemental table 3: Neonatal characteristics of neonates investigated for suspected late onset sepsis who were sampled versus those who were not , CRIB II: Clinical Risk Index in Babies II, nSOFA: neonatal Sequential Organ Failure Assessment, VIS: Vasomotor inotropic score, ROP: Retinopathy of prematurity, BPD/CLD: Bronchopulmonary Dysplasia/Chronic Lung Disease. Continuous data displayed with median values [Interquartile Range].
